# Rebuild the Academy: Supporting academic mothers during COVID-19 and beyond

**DOI:** 10.1371/journal.pbio.3001100

**Published:** 2021-03-09

**Authors:** Robinson W. Fulweiler, Sarah W. Davies, Jennifer F. Biddle, Amy J. Burgin, Emily H. G. Cooperdock, Torrance C. Hanley, Carly D. Kenkel, Amy M. Marcarelli, Catherine M. Matassa, Talea L. Mayo, Lory Z. Santiago-Vàzquez, Nikki Traylor-Knowles, Maren Ziegler

**Affiliations:** 1 Departments of Earth and Environment, Boston, Massachusetts, United States of America; 2 Department of Biology, Boston University, Boston, Massachusetts, United States of America; 3 School of Marine Science and Policy, University of Delaware, Lewes, Delaware, United States of America; 4 Department of Environmental Studies and Ecology & Evolutionary Biology, Kansas Biological Survey, University of Kansas, Lawrence, Kansas, United States of America; 5 Department of Earth Sciences, University of Southern California, Los Angeles, California, United States of America; 6 Marine Science Center, Northeastern University, Nahant, Massachusetts, United States of America; 7 Department of Biological Sciences, University of Southern California, Los Angeles, California, United States of America; 8 Department of Biological Sciences, Michigan Technological University, Houghton, Michigan, United States of America; 9 Department of Marine Sciences, University of Connecticut, Groton, Connecticut, United States of America; 10 Department of Mathematics, Emory University, Atlanta, Georgia, United States of America; 11 Department of Biology and Biotechnology, University of Houston-Clear Lake, Houston, Texas, United States of America; 12 Rosenstiel School of Marine and Atmospheric Sciences, University of Miami, Miami, Florida, United States of America; 13 Department of Animal Ecology and Systematics, Justus Liebig University Giessen, Giessen, Germany

## Abstract

The issues facing academic mothers have been discussed for decades. Coronavirus Disease 2019 (COVID-19) is further exposing these inequalities as womxn scientists who are parenting while also engaging in a combination of academic related duties are falling behind. These inequities can be solved by investing strategically in solutions. Here we describe strategies that would ensure a more equitable academy for working mothers now and in the future. While the data are clear that mothers are being disproportionately impacted by COVID-19, many groups could benefit from these strategies. Rather than rebuilding what we once knew, let us be the architects of a new world.

## Abstract 2

The COVID-19 pandemic is highlighting the many long-standing inequalities that academic mothers face. This Essay describes solutions for a more equitable academia, now and in the future, maintaining that rather than rebuilding what we once knew, we should be the architects of a new world.

## Introduction

Coronavirus Disease 2019 (COVID-19) has disrupted the academic system globally and exposed long-standing inequities and vulnerabilities of systematically marginalized groups in the academy. Recent studies [1,2] highlight the uneven toll the pandemic has taken on womxn scientists, and womxn of color in particular, who are parenting while also engaging in multiple academic duties (e.g., teaching, research, service). We applaud the data-driven research documenting this phenomenon, and welcome studies that help fill the data gap on womxn writ large (e.g., [3]). That said, to womxn across the academy, the toll COVID-19 is taking on their scientific productivity as well as their mental health comes as no surprise. It is simply the manifestation of a system that was not built by or for womxn in general, and mothers in particular [4]. The global COVID-19 pandemic will alter our society permanently. Failure to address equity now will undermine decades of work and progress made in bringing academic departments, especially Science, Technology, Engineering, and Mathematics (STEM) fields, closer to gender parity. In the spirit of the well-worn adage “*never let a good crisis go to waste*,” we propose using these unprecedented times as a springboard for necessary, substantive, and lasting change.

We fully acknowledge that this pandemic has negative, potentially severe, consequences for people of all groups (e.g., single or partnered, with or without children, inside or outside of academia, etc.). This essay is not meant to suggest that the toll of this crisis is restricted to female academics with children alone. We focus on mothers because we can speak to this personally, and because the impacts of COVID-19 within academia have been shown to be disproportionately greater on mothers (S1 Text). We believe it is a privilege to be a mother, and this essay is not meant to diminish the validity of the many womxn who are also dealing with infertility, family planning decisions, adoption, or pregnancy loss, while also navigating academia amidst a global pandemic. We recognize the diverse lived experiences of all those who identify as mothers in the academy and use maternal terminology (female, womxn, mother) with the intent of including the full spectrum of female and maternal identities.

Further, COVID-19 has laid bare the systemic racism that pervades academia and our society more broadly [5,6]. The pandemic has created additional disparities for mothers of color (MOC)—in particular black mothers—who, along with other black, indigenous, and people of color (BIPOC), have experienced disproportionately higher rates of unemployment, hospitalization, and death [7,8]. BIPOC faculty were already underrepresented in tenured and tenure-track STEM careers [9,10] with higher proportions of these faculty employed as lecturers and clinical faculty—positions which typically do not have tenure and are renewed annually. These nontenured faculty are now more vulnerable to losing employment during times of deep budget cuts [11]. Additionally, since the recent racial reckoning sparked by the killings of George Floyd and Breonna Taylor, conversations and emotionally charged service activities to confront systemic racism in academia have been assigned to or taken on by BIPOC scholars. This has placed additional burdens on MOC beyond the emergency childcare, teaching, research, and scholarship already required and expected of other academic mothers during COVID-19.

Ultimately, there is much work to do to foster a more equitable and inclusive environment for taking care of our families and ourselves while also pursuing research that will protect and preserve our planet and future generations. Here we provide concrete solutions to implement across multiple levels of the academic hierarchy that will help academic mothers in the sciences through this crisis, while simultaneously creating long-term change for a more equitable and inclusive academy. We are confident that these strategic actions can help solve the myriad problems mothers face (S1 Text). Further the solutions proposed here may help all scientists juggling personal issues as well as child- and/or elder-care while also trying to succeed in academia and beyond.

### Potential solutions for retaining mothers in science during and after COVID-19

**Fig 1:**
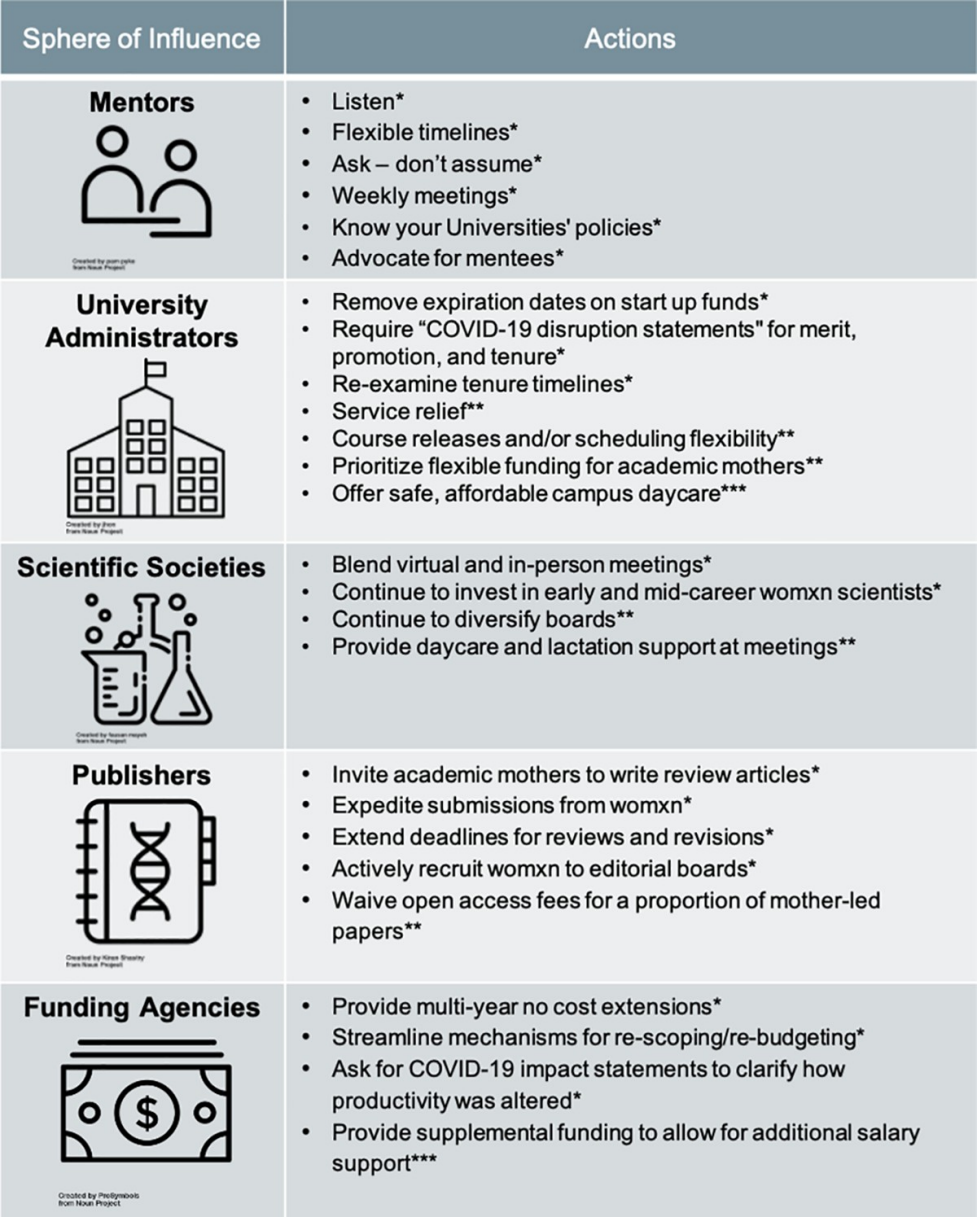
Overview of the proposed strategies to help support academic mothers through COVID-19 and beyond. These same strategies may also help others (e.g., fathers and elder-care givers). Other unsupported groups may benefit from these strategies and may also need additional strategies. Numbers of asterisks indicate relative fiscal requirements (* = low, ** = moderate, *** = high). The “mentor” icon is by pam pyke, the “college” icon is by jhon, the “science” icon is by fausan mayeh, the “journal” icon is by Kiran Shastry, and the “money” icon is by ProSymbols; all icons are from thenounproject.com.

Many of our suggestions require a monetary commitment. During times of increasing financial uncertainty, it may appear unwise to reallocate funding. However, scientists and academics have “talked” for a long time about how to stop the “leaky pipeline”[12]. Womxn, in particular BIPOC womxn, already bear the penalty of the rigid, unchanging incentive and evaluation practices of academia. COVID-19 is only compounding structural issues that already exist. Yet, few of us are in positions of power to change this system that we did not create. Adding more womxn to the system is not enough. We need men and womxn in power to acknowledge these issues and commit resources to fixing them. Universities can seek alumni donations to specifically fund strategies to retain womxn in science. Imagine, instead of having a name on a building, what if a donor could support initiatives for academic mothers? Much can and should be done (Fig 1).

Before discussing targeted strategies, we highlight universal infrastructure everyone should be fighting for: affordable, high-quality, childcare. Research has shown that when high-quality care is provided, mothers can and do reenter the workforce and children benefit [2,13,14]. Thus, this type of assistance makes both ethical and economic sense. Universities should provide on-campus daycare, or subsidies for off-campus daycares, as well as funds for additional childcare support when options are limited by social distancing restrictions. Universities with childcare facilities should also prioritize safety, including high frequencies of COVID-19 testing and sanitization practices. These resources should be made available to faculty, as well as staff, postdocs, and graduate students who are facing the same challenges on much lower salaries. We stress that all of us—regardless of academic position—can push for this goal, and those in power should be working tirelessly to do so.

### Advice for mentors of womxn with childcare duties

The first step to supporting mentees who become or are parents happens before they share their news or join your laboratory—**make it clear that you are wholly supportive of all personal choices and lifestyles, including family units with or without children, and that you value and also strive to achieve a healthy work-life teeter-totter** [15]. If you are an academic mother, there is no better example for your mentees than learning from your experiences. Being a good mentor means listening to your mentees and supporting their goals, and this becomes increasingly true when your mentees become parents. Your support can be especially important when they disclose they are expecting. Your reaction to this news sets the stage for how they will feel asking for what they need throughout their pregnancy, maternity leave, and return to campus. This is an emotional conversation even without the backdrop of a pandemic and keeping clear lines of communication open is essential. We recommend having weekly meetings that cover topics beyond research such as mental health/self-care.

For mentors of MOC, it is especially important to recognize the monumental additional mental burdens they carried even before the pandemic. Many black mothers worry about their children’s safety due to structural racism and violence against black children [16,17]. Additionally, many BIPOC mothers are already exhausted from daily microaggressions and question their belonging because they are often the only one of their race or ethnicity in their department or school/university, which leads to mental health declines [18]. COVID-19 has caused an increase in isolation of MOC who did not feel supported before the pandemic and already struggled with acceptance in a racist and sexist system.

#### Initiate discussions with your mentees about developing flexible timelines for both short- (e.g., lab work) and long- (e.g., graduation date) term goals in such a way that planning is viewed as positive, proactive, and supportive

If possible, finding ways to shift time-consuming tasks may allow graduate and postdoctoral mentees to focus their limited time on tangible products critical to current and future career stages and keep research moving forward. For example, outsourcing sample analysis or hiring technicians to complete field/lab work could help meet the demands of a research project while mentees learn to juggle school and work with childcare. If mentoring a postdoctoral associate, Principal Investigators (PIs) should work with them to apply for bridge funding.

It is also critical to **keep mentees with childcare responsibilities involved in lab interactions, departmental activities, and multi-institution collaborations.** Be conscious of not deciding whether to include/exclude mentees based on perceived availability, stress, or interest. Consider including them in collaborative projects that provide networking opportunities and the potential to learn new skills. In addition, encourage mentees to find a peer group to discuss the unique challenges of navigating academia during a pandemic while raising children. A peer group [19] can provide a safe and supportive environment for idea exchange and discussion [20].

Finally, **mentors need to be familiar with institutional parental leave policies in order to help mentees navigate options and make informed decisions.** In many cases, details of parental leave policies are missing critical information [21], such as where funding comes from for paid leave. Clarifying and addressing these policy gaps ahead of time can reduce stress for mentors and mentees and prevent mentors from trying to navigate nebulous policies, which might be particularly difficult during COVID-19 when colleges and universities are concerned about short- and long-term financial stability. If paid leave is not available, mentors should advocate for policy change and explore alternative solutions in the interim (e.g., leave donation).

### Advice for university administrators

University administrators, including Department Chairs, Deans, and Provosts, wield enormous power over how faculty, students, and staff navigate parental duties under normal circumstances through the establishment and enforcement of policies and procedures. While it feels that every policy has been rewritten to account for COVID-19, university administrators have a responsibility to advocate and champion the types of strategies suggested below in order to mitigate the disparate effects of COVID-19 on working mothers specifically and parents more generally. However, **administrators should not make gender- or race-neutral policies because the effects of the pandemic are not neutral across race or gender**. Such policies will fail to bring about equity, similar to what has been seen with gender-neutral parental leave [13,22]. Given the large uncertainties around the frequency and duration of COVID-19 disruptions, any changes to current policies or new policies implemented will need greater flexibility, particularly around reappointment, tenure, and promotion. The solutions described below include those first suggested by the 500 Women Scientists [23] and a letter to the University of Wisconsin Administration [24].

Departmental chairs at research-intensive institutions should **allocate flexible funds to support research productivity of academic mothers, in particular BIPOC mothers.** Research productivity of faculty is directly correlated to research productivity of their graduate students and postdocs. Thus, flexible funds could buy-out graduate student teaching requirements, especially if that student has childcare duties and/or is mentored by a woman with childcare duties. This will allow for research progress to continue, even during COVID-19. Similarly, funding for parental leave for graduate students and postdocs should not be the burden of the primary investigator. While we wait for leadership at the federal level, grant agencies and university administrators must provide funding for basic leave in order to ensure that the hiring of womxn scientists is not disincentivized.

Department chairs at teaching-intensive institutions should lessen teaching loads **by providing course releases or ensuring no new course development.** In addition, administrators can facilitate collaborations with internal or external partners that can aid in the continuation of research activities. A faculty member may be able to offer their expertise, allow access to equipment and/or facilities, and help manage a research program, potentially remotely. Faculty should be required to describe the special circumstances and challenges faced while teaching in a pandemic in their annual review and/or evaluation statements.

Many universities have offered voluntary or automatic 1-year blanket tenure extensions in an effort to support pretenure faculty during COVID-19. These extensions are offered in good faith; however, previous work has shown that tenure clock extensions are viewed negatively during tenure evaluation [25] and fail to level the playing field between mothers and fathers (i.e., maternity/paternity leave; [22]). Given that academic mothers have likely already had at least 1 tenure clock extension, further extensions could impose additional penalties and exacerbate financial inequalities that already exist between male and female academics [26]. This is particularly true when tenure evaluators don’t have clear instructions for evaluating “stoppage time” [25]. One solution may be to **include only a fixed number of performance years in tenure dossiers for evaluation** [27]. Another solution is working to adjust expectations and requirements for the traditional tenure period, rather than prolonging the tenure process. It is already known that womxn are PIs on fewer grants [28,29], are less likely to be published in high-ranking journals [30], are cited less [31,32], and are evaluated more harshly in teaching evaluations and reference letters [33,34]. Racial bias further exacerbates these issues for womxn of color [35]. And yet, we still embrace this model that assesses faculty based on H-indexes, journal rankings, and biased teaching feedback. Now is the time to develop a more holistic assessment approach that values interdisciplinary research, broader impacts, efforts to improve group and departmental culture, and diversity, equity, and inclusion efforts (i.e., [36]).

Not all research programs face the same obstacles during the pandemic. Shutdowns and reopenings are highly geographically variable. This variability in disruption will affect peer comparisons during tenure/promotion review processes and may create additional obstacles to retaining womxn. **We recommend that all institutions incorporate a “COVID-19 Disruptions” statement in tenure and promotion files with explicit instructions for external reviewers and internal review committees to consider inequalities generated by the pandemic**. These statements should be a required component of all tenure dossiers, even those of faculty who have not been heavily impacted by the pandemic. Templates should be provided with clear instructions and objective criteria for limiting biases that can arise when evaluating tenure packages [25]. Coupled to this, **we propose that all early career womxn scientists with childcare responsibilities be relieved of departmental/university service requirements for the duration of this pandemic**. An explanation of this service release should explicitly state that this lack of service is not to be held against them during merit and promotion review. Universal instructions should also be supplied to any external evaluators so that they are aware of how the University is handling this statement and any associated tenure clock extensions. Malisch and colleagues [13] provide an excellent set of questions or evaluation committees to consider. Other members of the department/university community may choose to pick up additional service to help fulfill these service needs, and their efforts should be specifically highlighted in their tenure/promotion or merit evaluations.

#### Startup expiration dates should be removed or extended to tenure receipt

Removing time constraints on startup funds allows womxn to make financial decisions about their labs within a time frame that works for their situation. Even better, Universities can provide support to offset “lost start-up” in the form of Research Assistant (RA) or technician salaries, and their associated fringe expenses, that PIs continued to pay throughout lab shutdowns. These funds could initially come from fundraising efforts from alumni or diversity, equity, and inclusion funds within the larger administration, and be built into university budgets going forward to support those mothers who will face ongoing challenges, specifically womxn of color.

Research productivity during COVID-19 will undoubtedly negatively affect and delay the Associate to Full Professor promotion of midcareer academic mothers. The challenges post-tenure mothers face are largely ignored and it is often assumed since they are tenured, delay and additional service requirements are “OK.” Even before the pandemic, womxn in STEM fields were underrepresented as Full Professors [37], and the road to Full Professor often lacks clarity [38]. Because these womxn are the most likely to assume leadership roles, and thus help change the broken academic system, we must ensure their timely promotion. **Deans and Provosts need to provide clear promotion guidelines and policies while also including specific COVID-19 policies to minimize delays to promotion. The strategies outlined above for early career mothers would also benefit promotion of midcareer mothers.**

### Advice for scientific societies

Scientific societies were reluctant to embrace virtual conferences because they pose significant logistical challenges and do not provide the same in-person interaction as face-to-face conferences. Yet disability and diversity advocates have long argued that virtual meetings have the opportunity to reach larger audiences and promote inclusion [39]. The sudden shift to virtual meetings has allowed many societies to experience the benefits and work through logistical challenges. These lessons should be leveraged to increase accessibility now and beyond. **Societies should consider how to retain elements of virtual meetings and blend them with traditional meeting schedules when in-person conferences resume.** Options such as prerecorded lectures and flexible start times will be especially helpful for individuals who are primary caretakers. **Societies should also consider how to keep registration fees for virtual participants as low as possible to encourage participation.** Finally, now more than ever, it is important to prioritize womxn, especially BIPOC mothers, to give plenaries and invited talks at virtual meetings.

**Scientific societies should also continue and expand efforts to diversify their governing boards, with special attention to include BIPOC womxn**. Societies must recognize that COVID-19 poses an overwhelming challenge to working mothers and caregivers but must not allow this to prevent recruitment into leadership positions important for career advancement and promotion. Governing boards should continue to recruit diverse candidates and mentor new governing board members. Because of the instability and shifting schedules associated with childcare and work during COVID-19, virtual and/or asynchronous communication for society business would provide much needed flexibility.

Some **scientific societies invest in early career members, especially BIPOC womxn, through research awards, travel grants, or publication funds to offset costs associated with publishing in their journals.** Societies without these support mechanisms should consider implementing them to offset the effects of COVID-19 on mothers. All societies should consider ways these programs can be adapted to promote mothers and/or primary caretakers’ work regardless of career stage. With the disproportionate pressure placed on mothers during this pandemic, publishing rates for womxn are expected to decline [1,40]. Therefore, having more opportunities, with flexible due dates, and streamlined submissions could support mothers trying to be involved in their societies and publish their work.

**Enhancing networking opportunities** for mothers could be particularly fruitful. As we have experienced through preparing this article, many mothers with little free time can join forces for productive and impactful collaborations. Because shared experiences allow for empathy, support, and realistic expectations, especially under our current circumstances, society-supported symposia or networking activities that bring together mothers in related fields could generate new and innovative research collaborations that are supportive, satisfying, and more productive. Encouraging inclusion of a “COVID-19 impact statement” in applications for travel/research awards can help individuals highlight the ways the shutdown impacted their research and home situations to ensure that funds are distributed to meet the greatest needs [41]. Finally, considering flexibility in travel or research awards to be used toward childcare costs could offset the time and financial burden for mothers as they contribute to society activities and scholarship.

### Advice for publishers

One important metric of scientific evaluation is peer-reviewed papers. Within the first few weeks of this pandemic, manuscript submissions by female researchers to preprint servers across disciplines dropped significantly or increased less than their male colleagues [40,42,43]. This trend was also apparent for womxn-led medical studies related to this pandemic [44,45]. Due to the time lag of the publishing process, we expect these disparities will further increase throughout the course and aftermath of the pandemic. Publishers and editors are at the center of the publishing machinery and have an opportunity to counterbalance these long-term detrimental effects on equitable science. Measures already being considered include **expediting submissions from self-identified womxn and prioritizing them during the peer-review process** [46]. During the COVID-19 pandemic, many journals have extended their deadlines to return reviews and revisions. We advocate adopting this practice more broadly across journals and continuing extended deadlines for the foreseeable future.

Open access (OA) publications attract a broader audience and are more frequently cited [47]; however, OA fees are high. **OA fee waivers could be extended to a certain proportion of manuscripts led by mothers with childcare responsibilities during this pandemic**. To help implement these practices, journals could add a self-reporting feature on their submission pages where mothers could choose to identify themselves. Similarly, manuscript images on journal covers help to highlight research and efforts could be made to make studies by mothers more visible. Men dominate editorial boards [41,48] and they are still twice as likely to be invited to submit papers to journals that consequently have higher acceptance rates [30]. **Invite and incentivize womxn to join editorial boards and recruit them to apply for Editor-in-Chief positions. Editors can amplify female voices by inviting them to write review or preview articles, as these get higher citations rates and indicate that the author is well established in the field** [41,46]**. We urge journals to be sure these efforts include, and focus on, BIPOC mothers.**

### Advice for funding agencies

Analogous to the advice for Colleges and Universities, nuanced approaches will be needed to address the variable impacts of COVID-19 on the community and on academic mothers in particular who, even before the pandemic, were submitting fewer grants than their male colleagues [14]. Facilitating no-cost extensions is a clear, relatively easy fix given current research restrictions. However, it will be as important to create the flexibility in funding rules for multiple no-cost extensions due to the multiyear nature of the pandemic. **We recommend that agencies consider what steps can be taken to reduce the paperwork burden associated with sequential applications, which are disproportionately needed by PIs with additional care-giving roles.** A streamlined mechanism for proposing scope of work revisions and rebudgeting requests would allow academic mothers to focus efforts on progressing research. This will be most essential for in-progress awards where salary support for students and postdocs has continued while their ability to complete project objectives has been hampered, creating a budget imbalance. **Funding agencies could consider making supplemental awards during extensions to allow for additional salary support for grant-funded project personnel to facilitate completion of the original scope of work when research is able to resume safely**. If additional funds are available, agencies could also consider developing short-term bridge funding awards to support academic mothers at all career stages as mothers are more likely than fathers to leave full-time STEM positions due to increasing childcare needs [49].

Finally, agencies should consider how impacts to productivity during this period will reflect on future funding applications. For example, the National Science Foundation requires a succinct summary of results from prior funding support. **Incorporation of a COVID-19 Disruptions statement could facilitate a more objective assessment of publications and products**.

## Conclusions

This essay was inspired when, yet another study was published on how working scientist mothers are disproportionately impacted by the COVID-19 pandemic. We found ourselves confronted with the realization that the academy was publishing data-driven science showcasing our lived experiences as academic mothers, but that these studies mostly highlighted the problems and not potential solutions. Studies quantifying problems are important because they provide data that can be leveraged to raise awareness of issues we experience in our careers, both in the past and now during these exceptionally challenging times. However, data do not solve problems. We need actionable solutions. Here, we described a series of strategies across spheres of influence that can help reduce the burden of this pandemic on mothers in science. As working mothers, we cannot solve this problem or implement these solutions alone [12]. We implore our universities, societies, and scientific leaders to carefully consider where and when they could leverage their power to implement the solutions presented here. Importantly, we urge leaders not to forgo action simply because a solution did not work before. Such a failure could be due to “false fails”—i.e., the strategy was not implemented correctly, for long enough, at the right time, etc. (Table 1). Ultimately, science and our world will benefit when there is equity for womxn and mothers.

**Table 1:** Creative solutions should be applied to help mothers through COVID-19 and beyond. A variety of solutions should be implemented, even if in the past they did not work as previous failures might be “false fails” due to poor timing, roll-out, communication, etc.

Problem	False Fail	Proposed Solution
Lack access to high-quality, affordable childcare	It’s too expensive to provide daycare	University provides daycare. Subsidize daycare on a sliding scale—with free daycare for graduate students, discounted daycare for postdocs, assistant professors.
Teaching requires significant time and mental space, resources that are at a minimum right now	We don’t have resources to hire new faculty to teach	Ensuring no new course development or hire postdoctoral teaching fellows
Decreased attendance at conferences	Virtual conferences are not as fun, we miss one-on-one interactions	Provide conferences in multiple modalities
Women underrepresented on Editorial Boards	Midcareer women turn down invitations to be editors due to time constraints, but men accept invitations; underrepresentation of women too hard to change.	Talk with women you invite to help brainstorm how they could participate on an editorial board. For example, could they decrease their departmental/university service level? Is it possible for them to handle fewer manuscripts? Are there incentives that could make picking up this new service more appealing (e.g., 1 free open access publication in the journal per year)
These solutions require money	Universities do not have money to implement these solutions	Universities are able to raise money for new sports arenas, buildings, and individual labs/projects. Why not for keeping women in science?

## Supporting information

S1 Text Here we provide an extended description of our motivation for writing this piece as well as more details on the negative consequences of COVID-19 on academic mothers. (DOCX)

## Abbreviations

BIPOC: black, indigenous, and people of color
COVID-19: Coronavirus Disease 2019
MOC: mothers of color
OA: open access
PI: Principal Investigator
RA: Research Assistant
STEM: Science, Technology, Engineering, and Mathematics
