# Supplementary material for: Rebuild the Academy: Supporting academic mothers during COVID-19 and beyond
Source: PLoS Biol. 2021 Mar 9;19(3):e3001100. doi: 10.1371/journal.pbio.3001100 (PMC7942998; doi:10.1371/journal.pbio.3001100)
Supplement: S1 Text — (DOCX) [file pbio.3001100.s001.docx]

**S1 Text**

**Overview of Motivation to write this piece**

Investigators with young families, and especially female principal investigators (PIs), are experiencing negative impacts as a result of work from home policies coupled with ongoing childcare and school opening challenges and resultant homeschooling. For example, childcare is sparse or unavailable due to mandated physical distancing restrictions that have led to significant reductions in capacity at some facilities, and permanent closures of others. This has forced parents to keep children at home with limited/no back-up care options. Even as schools re-open, many schools are offering hybrid instruction, which limits uninterrupted windows for academic work. Moreover, many families cannot risk sending their children back to daycare or school for fear of infection and face the prospect of having their children schooled from home, via virtual instruction or traditional home-schooling. Parents, and in particular womxn, are tasked with facilitating their children’s learning and enrichment coupled with the expectation to maintain research productivity and teaching excellence, often without outside assistance. Notably, research suggests that even in dual career households, mothers often perform a greater proportion of child care and household duties when compared to fathers [1,2] and, while new parents are more likely to leave full-time STEM jobs than their childless peers, new mothers are twice as likely to leave STEM as new fathers [3].

The current childcare and schooling situation is especially hard on pre-tenure mothers who struggle to bolster their scientific productivity while also remaining engaged with their family. That said, mothers at all career levels with children or elders at home may be disproportionately affected by policies associated with COVID-19. We fear an increase in tenure denials related to COVID-19, as well as a decrease in promotions to Full Professor, thereby continuing to limit the number of womxn advancing into leadership positions within the academic pipeline [4–6]. Of course, delays in career advancement also apply to those not on the tenure track and may be especially difficult for academic mothers in adjunct or lecturer positions, which are more likely to be in jeopardy due to the financial ramifications of COVID-19.

**Consequences of the COVID-19 pandemic on research for academic mothers**

Although the general consequences of COVID-19 on academia are substantial and have been discussed in detail (e.g.,[7]), Biological and Environmental Sciences (BES) research programs may be especially impacted due to an emphasis on long-term datasets, fieldwork, and large-scale laboratory and field experiments that require teamwork. Of course, the impact of the COVID-19 pandemic on research productivity will be largely dependent on the type of research conducted in each lab and each investigator’s personal responsibilities. The effects of lab shutdowns are especially difficult for empirical or experimentally-focused researchers. These impacts are even more severe for those labs conducting research that requires fieldwork, which has become largely intractable during the pandemic due to loss of access to field sites, cancelation and/or bans of domestic and international travel, and limitations on the number of field researchers due to physical distancing requirements. The long-term consequences of delayed/canceled field seasons are more likely to negatively influence mothers due to childcare responsibilities. As travel and field sites reopen, mothers without access to childcare will need to prioritize childcare responsibilities and will remain unable to travel, ultimately extending the consequences of COVID-19. Even as childcare facilities reopen in their region, it may be too risky to dedicate time to planning and conducting field work - especially at remote locations - due to fears of future campus or field site shutdowns, which would again render fieldwork impossible and potentially make community and/or family members sick.

Junior members of the research community, which are more likely to be womxn [8], will be hit hardest because projects and graduate students are more likely to be in early phases. As a consequence, junior investigators are often left focusing on literature reviews or completing legacy datasets from their previous positions. In addition, because many womxn choose to wait to start their family until after they find a permanent academic position [5], these consequences are again more likely to have a larger impact on academic mothers. Lastly, physical distancing restrictions have limited the number of researchers in labs and will therefore impose greater limitations on PIs with small lab spaces, which are again most likely to be female early-career PIs [9].

Publications are the currency in academia; however, the publication process itself has experienced upheaval due to COVID-19 [10–13]. In general, the process has slowed significantly, from obtaining reviews to receiving proofs, which decreases publication rates and has implications for resulting citation rates. In addition, editors are reporting surges in manuscript submission that are disproportionately led by male authors [10,14], and this influx in submissions is likely to lead to increased competition for already limited space in journals. While many academic mothers have been able to perform some level of analysis and manuscript drafting during the pandemic, the pace of publication and subsequent citation rates will inevitably experience greater lag times. This will only be compounded in fields that require multiple year data sets or extensive fieldwork. Child rearing during this global pandemic has left academic mothers with even smaller units of time, which makes deep intellectual thought and writing, which are required for both publications and grants, next to impossible. These sorts of hurdles have specifically left mothers publishing significantly less than their colleagues without children [15].

The abrupt transition to online teaching in spring 2020 presented a new challenge for all academics. These challenges were especially felt by womxn caring for young children and/or leading the at-home learning requirements for children. Now, many institutions are moving to a hybrid model of teaching for fall, essentially doubling the course preparation required at a time when many womxn remain child caregivers at home. Similarly, womxn graduate students with childcare responsibilities and who serve as teaching assistants (common practice in the BES field) now have to spend more time re-developing course materials to complement online learning needs, which detracts from time spent on their dissertations.

Grant deadlines have not been significantly altered and we anticipate significant changes may be made to federal funding rates and priorities as a result of the pandemic and associated economic fallout. It seems inevitable that institutional internal grants will be slashed with impending budget cuts, and likely that state and federal grant agencies will be restricting funds in order to deal with financial repercussions of COVID-19. While these cuts will affect all academics, academic mothers who are also balancing childcare responsibilities will not have the bandwidth to submit grants in hopes of getting funded before these looming COVID-19 cuts are made. These issues are compounded by the fact that womxn at all career stages were already applying for grants at lower rates [8]. Because the grant review cycle takes approximately 6 months, missing deadlines now means less future funding. This decrease in grant applications by mothers will perpetuate the impacts of the pandemic for years to come. For some, it may result in insufficient funding to continue in science altogether.

Travel restrictions and the associated loss of networking opportunities has implications for initiating collaborations, demonstrating research productivity, and connecting with potential external review evaluators. A massive number of in-person networking opportunities have evaporated through cancelled regional, national, and international meetings and seminar invitations, and reduced numbers of seminar speakers visiting campuses. By necessity, many of these opportunities have moved to an online format. Ironically, many organizations are touting this as an opportunity for increased inclusion, under the auspices that online events can be attended by any and all who are interested. That presumption is not true, as womxn at home who have childcare obligations are hindered in participating because they may not have the physical space or time to do participate in professional activities. Further, many womxn feel that they also do not have the mental and emotional space to prepare and present a research seminar under the current circumstances. Declining these opportunities decidedly impacts their careers as networking is a principle means of generating career opportunities such as new collaborations and job offers. Plus, the connections made during these visits often result in offers of reviewers of promotion and tenure packets, thereby potentially limiting the dissemination and recognition of early career scientists. Finally, annual merit review as well as tenure and promotion packages keep track of these seminars as a way to assess how faculty are regarded in their field.

Lastly, the long-term effects on the productivity and mental health of graduate student mothers remain unclear. While substantial focus is understandably being given to late-career Ph.D. students and postdoctoral researchers facing immediate funding and job market challenges, the younger cohorts must also be considered. For some postdocs and graduate students, child rearing has now been compounded on top of these already leaky stages in the academic pipeline [16] that require intense levels of productivity. Clearly, the COVID-19 pandemic has the potential to make an already leaky pipeline burst, especially when it comes to academic mothers. People in positions of power now have the opportunity and responsibility to ensure that this does not occur.

**References**

1. Craig L. Does father care mean fathers share? A comparison of how mothers and fathers in intact families spend time with children. Gend Soc. 2006;20: 259–281.

2. Sallee, M., Ward, K. and Wolf-Wendel L. Can Anyone Have it All? Gendered Views on Parenting and Academic Careers. Innov High Educ. 2016;41: 187–202.

3. Cech EA, Blair-Loy M. The changing career trajectories of new parents in STEM. Proc Natl Acad Sci U S A. 2019. doi:10.1073/pnas.1810862116

4. Bonawitz M, Andel N. The Glass Ceiling Is Made of Concrete: The Barriers to Promotion and Tenure of Womxn in American Academia. Forum on Public Policy Online. 2009.

5. Goulden M, Mason MA, Frasch K. Keeping Womxn in the Science Pipeline. Ann Am Acad Pol Soc Sci. 2011. doi:10.1177/0002716211416925

6. Staniscuaski F, Reichert F, Werneck FP, de Oliveira L, Mello-Carpes PB, Soletti RC, et al. Impact of COVID-19 on academic mothers. Science. 2020. doi:10.1126/science.abc2740

7. Malisch JL, Harris BN, Sherrer SM, Lewis KA, Shepherd SL, McCarthy PC, et al. Opinion: In the wake of COVID-19, academia needs new solutions to ensure gender equity. Proceedings of the National Academy of Sciences of the United States of America. 2020. doi:10.1073/pnas.2010636117

8. Rissle R. LJ, Hale KL, Joffe NR, Caruso NM. Gender Differences in Grant Submissions across Science and Engineering Fields at the NSF. Bioscience. 2020.

9. Acton SE, Bell AJ, Toseland CP, Twelvetrees A. A survey of new PIs in the UK. Elife. 2019. doi:10.7554/eLife.46827

10. Flaherty C. No Room of One’s Own. Insid High Ed. 2020.

11. Andersen JP, Nielsen MW, Simone NL, Lewiss RE, Jagsi R. COVID-19 medical papers have fewer womxn first authors than expected. Elife. 2020. doi:10.7554/eLife.58807

12. Muric, G., Lerman, K. and Ferrara E. COVID-19 amplifies gender disparities in research. arXiv Prepr arXiv200606142. 2020.

13. Myers KR, Tham WY, Yin Y, Cohodes N, Thursby JG, Thursby MC, et al. Unequal effects of the COVID-19 pandemic on scientists. Nature Human Behaviour. 2020. doi:10.1038/s41562-020-0921-y

14. Frederickson M. COVID-19’s gendered impact on academic productivity. Available: https://github.com/drfreder/pandemic-pub-bias/blob/master/README.md

15. Edwards, S.M. & Snyder L. Yes, balancing work and parenting is impossible. Here’s the data. Washington Post. 2020. Available: https://www.washingtonpost.com/outlook/interruptions-parenting-pandemic-work-home/2020/07/09/599032e6-b4ca-11ea-aca5-ebb63d27e1ff_story.html

16. Lee L. just the facts ma’am: the gender gap in academia, from the classroom to the bank. FASEB J. 2015.
